# Efficient and Scalable Process to Produce Novel and Highly Bioactive Purified Cytosolic Crystals from Bacillus thuringiensis

**DOI:** 10.1128/spectrum.02356-22

**Published:** 2022-08-10

**Authors:** Jeffrey Chicca, Nicholas R. Cazeault, Florentina Rus, Ambily Abraham, Carli Garceau, Hanchen Li, Samar M. Atwa, Kelly Flanagan, Ernesto R. Soto, Mary S. Morrison, David Gazzola, Yan Hu, David R. Liu, Martin K. Nielsen, Joseph F. Urban, Gary R. Ostroff, Raffi V. Aroian

**Affiliations:** a Program in Molecular Medicine, UMASS Chan Medical School, Worcester, Massachusetts, USA; b M.H. Gluck Equine Research Center, Department of Veterinary Science, University of Kentucky, Lexington, Kentucky, USA; c Department of Chemistry and Chemical Biology, Harvard University, Cambridge, Massachusetts, USA; d U.S. Department of Agriculture, Agricultural Research Service, Beltsville Agricultural Research Center, Animal and Parasitic Diseases Laboratory, Beltsville, Maryland, USA; e U.S. Department of Agriculture, Agricultural Research Service, Beltsville Human Nutrition Research Center, Diet, Genomics and Immunology Laboratory, Beltsville, Maryland, USA; University of Wisconsin—Madison

**Keywords:** *Bacillus thuringiensis*, crystal protein, purification, drugs, anthelmintic, parasites

## Abstract

Bacillus thuringiensis (Bt) is a Gram-positive soil bacterium that is widely and safely applied in the environment as an insecticide for combatting insect pests that damage crops or are disease vectors. Dominant active ingredients made by Bt are insect-killing crystal (Cry) proteins released as crystalline inclusions upon bacterial sporulation. Some Bt Cry proteins, e.g., Cry5B (formally Cry5Ba1), target nematodes (roundworms) and show exceptional promise as anthelmintics (cures for parasitic nematode diseases). We have recently described inactivated bacteria with cytosolic crystal(s) (IBaCC) in which bioactive Bt Cry crystals (containing Cry5B) are fully contained within the cytosol of dead bacterial ghosts. Here, we demonstrate that these IBaCC-trapped Cry5B crystals can be liberated and purified away from cellular constituents, yielding purified cytosolic crystals (PCC). Cry5B PCC contains ~95% Cry5B protein out of the total protein content. Cry5B PCC is highly bioactive against parasitic nematode larvae and adults *in vitro*. Cry5B PCC is also highly active *in vivo* against experimental human hookworm and *Ascaris* infections in rodents. The process was scaled up to the 100-liter scale to produce PCC for a pilot study to treat two foals infected with the ascarid *Parascaris* spp. Single-dose Cry5B PCC brought the fecal egg counts of both foals to zero. These studies describe the process for the scalable production of purified Bt crystals and define a new and attractive pharmaceutical ingredient form of Bt Cry proteins.

**IMPORTANCE** Bacillus thuringiensis crystal proteins are widely and safely used as insecticides. Recent studies have shown they also can cure gastrointestinal parasitic worm (nematode) infections when ingested. However, reproducible, scalable, and practical techniques for purifying these proteins have been lacking. Here, we address this severe limitation and present scalable and practical methods for large-scale purification of potently bioactive B. thuringiensis crystals and crystal proteins. The resultant product, called purified cytosolic crystals (PCC), is highly compatible with ingestible drug delivery and formulation. Furthermore, there are growing applications in agriculture and insect control where access to large quantities of purified crystal proteins is desirable and where these methods will find great utility.

## INTRODUCTION

Bacillus thuringiensis (Bt) is a naturally occurring Gram-positive soil bacterium and globally the most successfully and commonly used biologically produced insecticide, comprising 75% of the biopesticide market and a major insecticide used in organic farming (1, 2). Bt strains have evolved that kill lepidopteran (caterpillars) and coleopteran (beetles) pests that are highly damaging to agriculture and to dipteran (mosquitoes, blackflies) pests that are important for vectoring major global diseases, like malaria, river blindness, and dengue fever (3–6). The main (but not only) insecticidal ingredients that Bt makes are three-domain crystal (Cry) proteins, so named because these proteins form large macromolecular crystalline inclusion bodies during bacterial sporulation (7). These proteins are safe and nontoxic to vertebrates, showing no effect levels in the >1,000-mg/kg of body weight range (8, 9). This safety factor has played an important role in the adoption and expansion of uses of Cry proteins, including as expressed proteins in transgenic crops, e.g., to produce insect-resistant corn and cotton varieties that are now planted in >108 million hectares worldwide (10). In addition to targeting insects, some Bt Cry proteins also target nematodes, including intestinal parasites of humans, dogs, horses, sheep, and pigs and parasites of plants (11–17). Because nematodes are important parasites of humans, animals, and plants and because of their excellent safety record and profile, there is significant interest in using Bt Cry proteins to control nematode parasites.

For environmental applications, Bt crystals have always been deployed along with the live spores that produce them (i.e., as live spore crystal lysates) (18, 19). Although the bacterium Bt has been considered safe, it is in the same family as Bacillus cereus (a known enteric pathogen) and displays and can express a similar repertoire of potential virulence genes as B. cereus does, and there are concerns that live Bt sprays can be related to sporadic food poisoning outbreaks (20–24). As such, in some places such as Europe, there are limits in terms of acceptable residual levels of Bt spores on foods sold (23, 24). There are also increasing environmental concerns with using live spores, e.g., Bt spore accumulation in nontarget beneficial insects (25). For these and other reasons, using live Bt spore crystal lysates would be challenging from regulatory and therapeutic vantage points as ingested anthelmintics (e.g., oral cures for intestinal nematodes) (14). The use of live spores also results in environmental contamination, with potential implications for the development of resistance, e.g., of parasitic larvae that live in the soil (14). For all these considerations, we developed a novel form of Cry proteins delivered inside of dead bacteria. This novel form is called inactivated bacteria with cytosolic crystal(s) (IBaCC) (14). With IBaCC, Bt crystals are formed during vegetative growth/stationary phase in sporulation-defective Bt. The Bt crystals are trapped in the cytosol of the nonsporulated *Bacillus* bacteria (14). Such bacilli cells are easily killed by food-grade essential oils, while at the same time nematode-active Bt crystals left inside their dead bacterial ghosts fully retain bioactivity (14).

While working with IBaCC, we found that the dead bacterial ghosts treated with essential oil became somewhat brittle, raising the possibility that a simplified protocol might be developed in which Bt crystals can be isolated to high purity apart from other bacterial components. Here, we describe such a simplified methodology for the isolation of purified cytosolic crystals (PCC). Cry5B PCC was tested for bioactivity *in vitro* and *in vivo* against parasitic nematodes. We also scaled up purification of PCC by utilizing a 100-liter culture of Bt Cry5B IBaCC. Use of Cry5B PCC from this scaled-up run for treatment of parasites in large animals (foals) is also presented.

## RESULTS

### IBaCC cells can be used to produce PCC.

**FIG 1:**
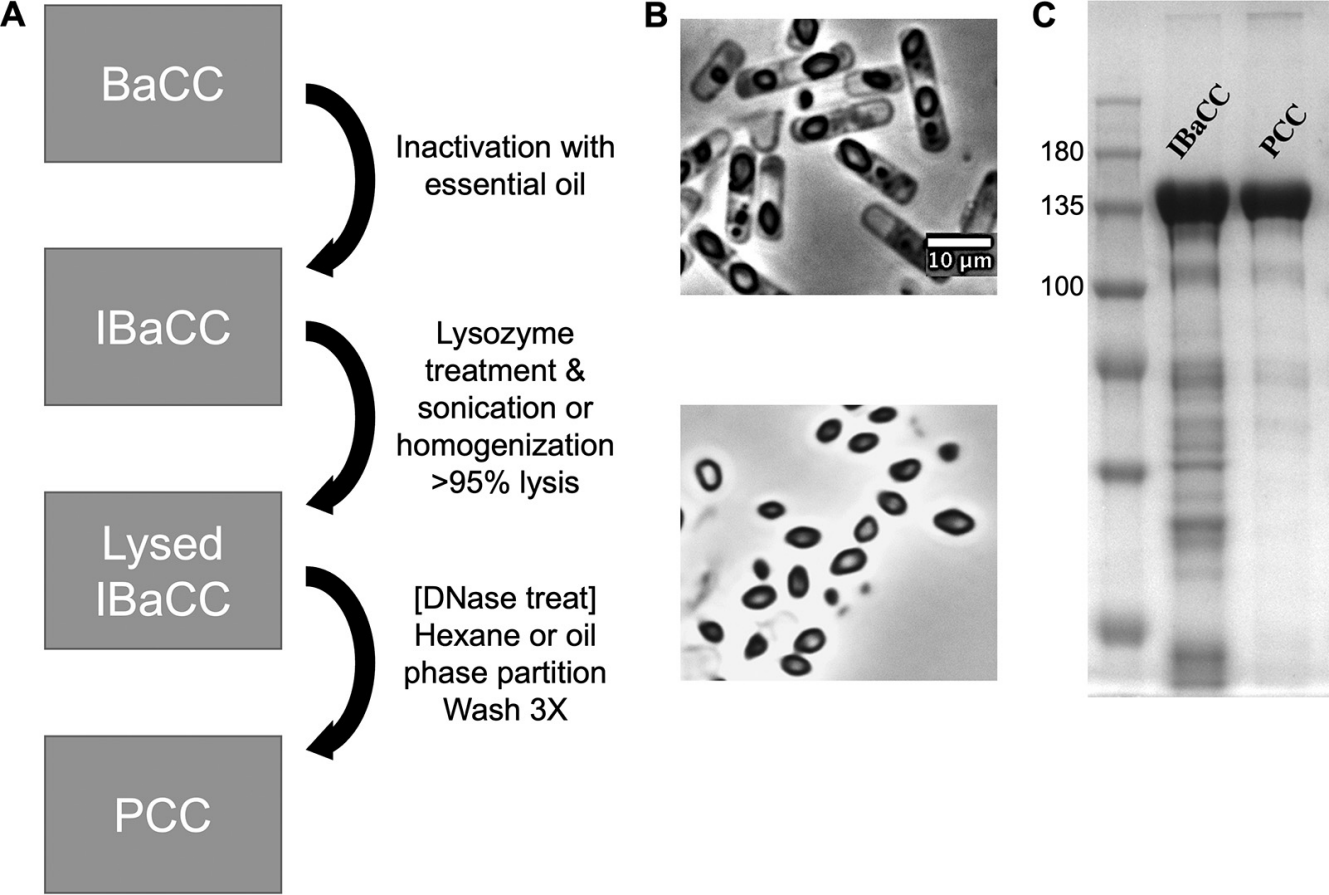
Purified cytosolic crystals. (A) A flow diagram of the process of turning BaCC into PCC. DNase treatment is optional. (B) A comparison of IBaCC (upper panel) and PCC (lower panel) as visualized by using an Olympus BX60 microscope with 100× oil objective. The scale is the same in both panels. (C) SDS-PAGE visualization of Cry5B IBaCC and PCC. The left-most lane includes protein standards (in kilodaltons).

We hypothesized that IBaCC cells could be cracked open and phase partitioning could be used to purify crystals away from soluble and insoluble bacterial debris. After significant trial and error, we developed a protocol that allowed for purification of crystals away from bacteria (Fig. 1A) (see Materials and Methods, below). We found that recombinant IBaCC cells (killed with essential oil) expressing Cry5B crystals were relatively brittle and efficiently lysed by the combination of lysozyme and homogenization. Removal of cellular debris was achieved by phase partitioning over hexane. An optional DNase treatment step was included to remove DNA fragments, if desirable (see Materials and Methods).

The result of these process steps gave rise to PCC, which was viewed by light microscopy (Fig. 1B). With sodium dodecyl sulfate-polyacrylamide gel electrophoresis (SDS-PAGE), a significant reduction in background protein gels was seen (Fig. 1C), with Cry5B making up 31% of Cry5B PCC by dry weight and 96% of the total protein. We were also able to make Cry1Ac PCC from insecticidal Cry1Ac protein (see below).

### Cry5B PCC is bioactive *in vitro*.

Blood-feeding hookworms are among the most important of all human parasitic infections (26, 27). Roughly half a billion people have hookworm infestation, leading to an estimated disease burden of 4 million disability-adjusted life-years (DALYs) and economic losses of US$139 billion/year from lost work productivity. Human hookworms fall into two nematode genera, *Necator* and *Ancylostoma*, both of which are sensitive to Cry5B IBaCC (14).

**FIG 2:**
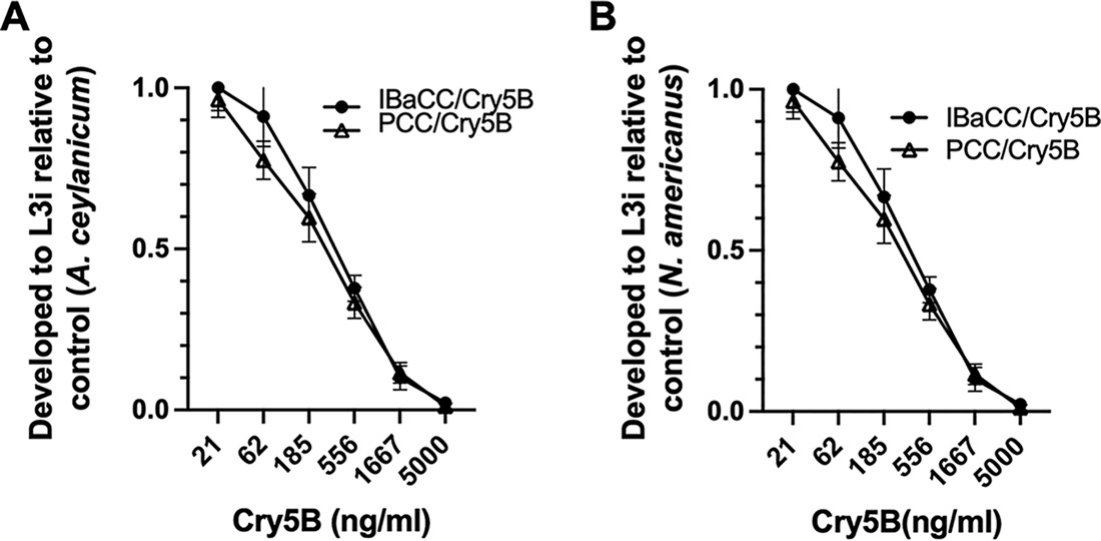
Comparison of the bioactivity of Cry5B IBaCC and PCC against developing hookworm larvae. (A) Ratio of *A. ceylanicum* hookworm eggs that developed to the L3i (infectious larval) stage in the presence of various doses of Cry5B presented as either IBaCC or PCC. (B) Ratio of N. americanus hookworm eggs that developed to the L3i (infectious larval) stage in the presence of various doses of Cry5B presented as either IBaCC or PCC. Data in both panels represent the averages of three independent trials. Error bars here and in all figures represent standard errors of the means.

We compared the bioactivity of Cry5B PCC and parent IBaCC against both genera of hookworm larvae using a larval development assay *in vitro* (28). Cry5B PCC potently inhibited Ancylostoma ceylanicum and Necator americanus hookworm larval development, with complete inhibition of development at 5 μg/mL (Fig. 2A and B). The efficacy with Cry5B was very similar to that of Cry5B IBaCC (Fig. 2A and B).

**FIG 3:**
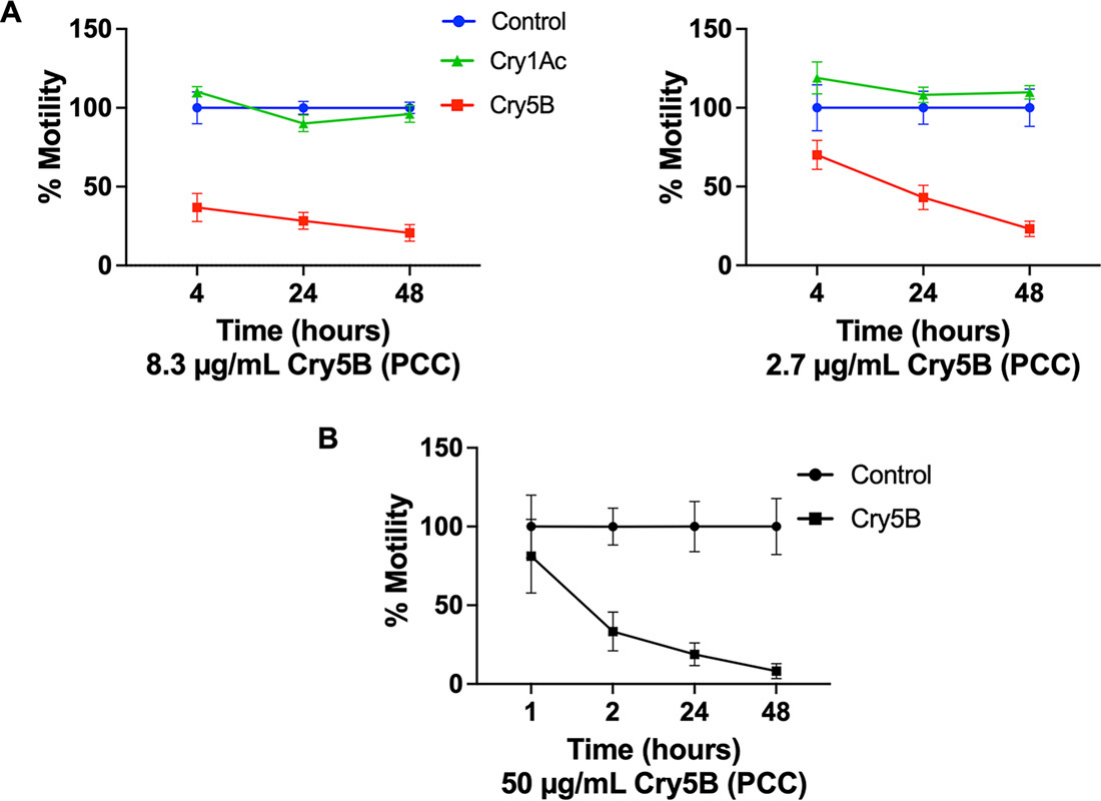
Intoxication of hookworm adults with PCC. (A) Adult *A. ceylanicum* hookworms were exposed *in vitro* to 8.3 μg/mL (left) or 2.7 μg/mL (right) Cry protein (Cry5B or Cry1Ac) as PCC. Motility was measured using the Worminator system (see Materials and Methods) and normalized to control (buffer only) at each time point. (B) Adult N. americanus hookworms were exposed to Cry5B PCC at 50 μg/mL, and motility was measured over time as described for panel A.

We also tested Cry5B PCC against adult-stage parasites *in vitro*. Cry5B PCC was found to be highly active *in vitro* against A. ceylanicum hookworm adults at two low concentrations. As a negative control for nonspecific activity of purified crystals, we also expressed and purified insect-active Cry1Ac PCC, which had no detectable activity against these nematodes (Fig. 3A). Good bioactivity of Cry5B PCC was also found against N. americanus hookworm adults (Fig. 3B).

### Cry5B PCC is bioactive *in vivo* against an experimental hookworm infection.

**FIG 4:**
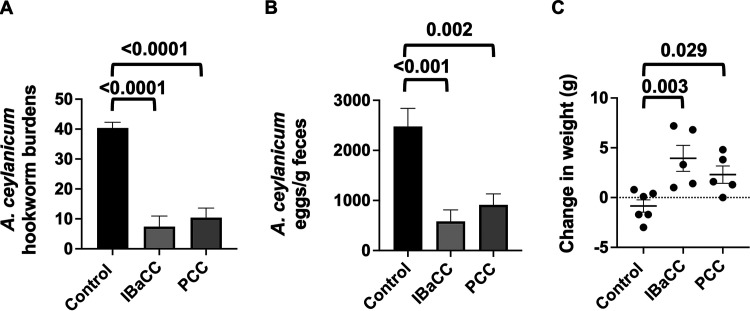
*In vivo* efficacy of Cry5B PCC against *A. ceylanicum* hookworm infections in hamsters. (A) Average intestinal hookworm burdens in hamsters treated with buffer, Cry5B as IBaCC (5 mg/kg), and Cry5B as PCC (5 mg/kg). Shown here and in other panels are *P* values for Cry5B treatment group comparisons with controls. *n* = 6 for the control group and *n* = 5 for each Cry5B-treated group. (B) Average fecal egg counts per gram of feces from the same hamsters as in panel A. (C) Average changes in weight from just before treatment until the end of the experiment, from the same hamsters as in panel A. Actual values are given in Table S1 in the supplemental material.

That Cry5B PCC was highly active against adult parasites *in vitro* suggested it might be efficacious against active parasitic infections *in vivo*. We therefore tested Cry5B PCC against an experimental hookworm infestation. Hamsters were experimentally infected with *A. ceylanicum*, a zoonotic parasitic hookworm that infects humans with reemerging importance (29, 30). Eighteen days after infection, when the *A. ceylanicum* hookworms reached fertile adulthood, the hamsters were split into three groups based on equivalent fecal egg counts (FECs). Via oral gavage, one group received buffer control, one group received a single dose of Cry5B IBaCC in buffer (final dose of 5 mg/kg of Cry5B per hamster), and one group received a single dose of Cry5B PCC in buffer (final dose of 5 mg/kg of Cry5B per hamster). Four days later, both parasite burdens and reproduction (fecal egg counts) were measured, as well as changes in weight from the day of treatment until the end of the study. Cry5B PCC and IBaCC treatments resulted in 74% and 82% *in vivo* reductions, respectively, in parasitic hookworm burdens relative to buffer control (Fig. 4A). The difference in efficacy based on parasite burdens between Cry5B IBaCC and PCC treatments was not statistically significant (*P* = 0.55). Cry5B PCC and IBaCC treatments also resulted in 63% and 77% reductions in parasite fecal egg counts, respectively (Fig. 4B). The difference in efficacy based on parasite fecal egg counts between Cry5B IBaCC and PCC treatments was not statistically significant (*P* = 0.33).

One of the sequelae of hookworm infection in humans is a negative impact on growth, which has also been seen in hamsters (31). We measured the change in weight over the 4 days between treatment and the end of the experiment (Fig. 4C). Both treatments, with Cry5B PCC or IBaCC, on average resulted in hamsters that showed significant weight gain (+2.3 g and +3.9 g, respectively) relative to that in the buffer control group, which showed weight loss (−0.85 g). The difference in weight gain between the PCC- and IBaCC-treated groups was not significant (*P* = 0.33).

**FIG 5:**
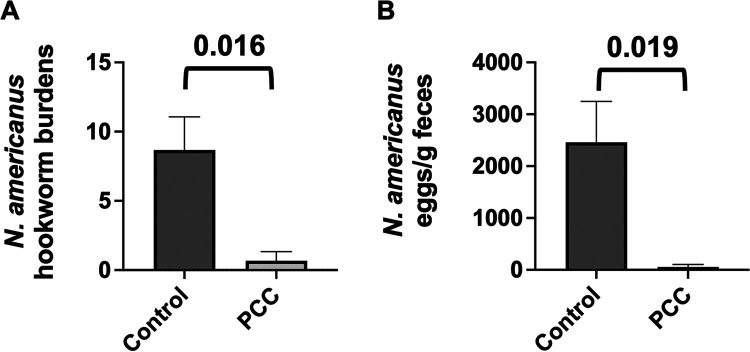
*In vivo* efficacy of Cry5B PCC against N. americanus hookworm infections in hamsters. (A) Average intestinal hookworm burdens in hamsters treated with buffer and Cry5B as PCC (10 mg/kg). *n* = 3 for both groups. (B) Average fecal egg counts per gram of feces from the same hamsters as in panel A. Actual values are given in Table S1 in the supplemental material.

A small-scale *in vivo* efficacy trial of Cry5B PCC was also tested against N. americanus hookworm infections in hamsters. N. americanus is the dominant hookworm parasite of humans worldwide (26). Cry5B PCC was also highly effective against this genus of hookworms, as a single 10-mg/kg dose given orally to infected hamsters resulted in a 92% reduction in hookworm parasite burdens relative to buffer controls and in a 98% reduction in parasite fecal egg counts (Fig. 5A and B).

### Cry5B PCC was effective against ascarids in a scale-up and pilot study in foals.

**FIG 6:**
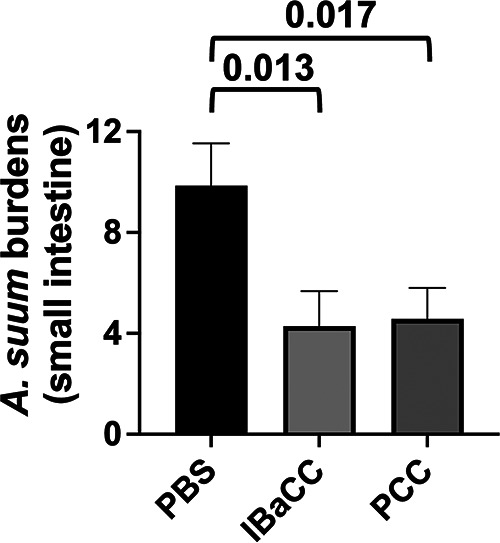
*In vivo* efficacy of Cry5B PCC against *A. suum* roundworm infections in mice. Average intestinal *Ascaris* burdens in mice treated with buffer and Cry5B as IBaCC (15 mg/kg) or as PCC (15 mg/kg). Shown here are *P* values for Cry5B treatment group comparisons relative to controls. *n* = 7 for each group. Actual values are given in Table S1 in the supplemental material.

Ascaris lumbricoides or intestinal large roundworm is one of the most common parasites of humans worldwide and highly related to, if not the same species as, Ascaris suum, the dominant intestinal nematode parasite of pigs (32–34). We have reported on an immunodeficient mouse model for *Ascaris suum* in which *A. suum* eggs administered via gavage to Th2-deficient mice developed to the early intestinal parasitic L4 stage, which could then be used for intestinal *Ascaris* studies in rodents (17). Here, we tested Cry5B PCC against this experimental infection (Fig. 6). A single 15-mg/kg dose of Cry5B PCC given via gavage led to a statistically significant 54% reduction in intestinal *A. suum* burdens in mice, similar to that of a single 15-mg/kg dose of Cry5B as IBaCC (57% reduction).

**FIG 7:**
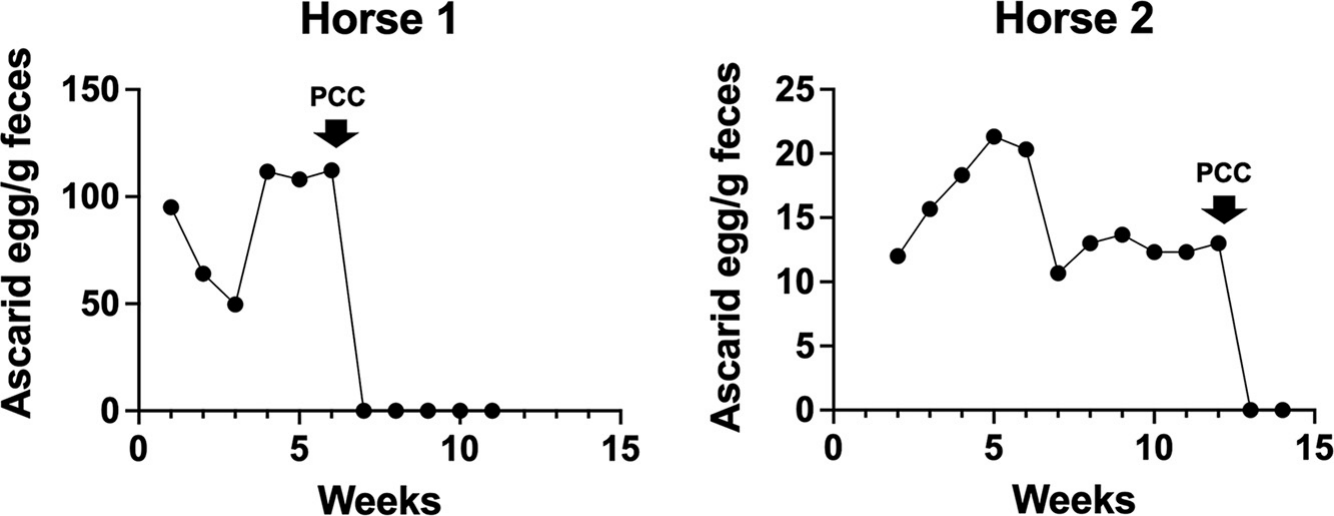
*Parascaris* fecal egg counts from two foals treated with scaled-up Cry5B PCC. Egg counts were taken weekly before and after treatment. Treatment occurred immediately after fecal samples were taken on the week indicated by the down arrow.

We previously demonstrated that Cry5B is effective in foals against infestations of *Parascaris* spp., a critical and potentially lethal parasite of young horses related to human *Ascaris* (17, 35). We performed a pilot study in foals infected with *Parascaris* spp. to see if enough bioactive PCC could be produced for a large animal trial. Cry5B IBaCC was produced on a 100-liter scale and then processed to PCC. Two foals, which had naturally acquired *Parascaris* spp. infections, were enrolled in the study. After 6 weeks of monitoring, one foal was pretreated with omeprazole to neutralize stomach acid and then given a single oral 10-mg/kg dose of Cry5B PCC (2.5 g Cry5B as PCC). The second infected foal was kept untreated as a control. One week later (week 7), the untreated foal retained its positive fecal egg count, whereas for the PCC-treated foal, fecal egg counts dropped to zero and stayed at zero for the duration of weekly testing (weeks 8 to 11) (Fig. 7). The following week (week 12), the untreated control foal that had retained positive ascarid fecal egg counts was pretreated with omeprazole and then given a single oral 10-mg/kg dose of Cry5B PCC. One week later (week 13), fecal egg counts dropped to zero for this foal and stayed at zero for the duration of testing (week 14) (Fig. 7).

## DISCUSSION

Here we have described a scalable method for purification of crystals from sporulation-defective B. thuringiensis. From a strain expressing Cry5B, the protein content in PCC was >95% Cry5B. Cry5B PCC had excellent bioactivity, as indicated by its potent *in vitro* effects on larval and adult stages of two species of human hookworms. Cry5B PCC was also highly effective at clearing two different species of human hookworms in infected rodents and two different species of ascarids, one in rodents and one in horses. As part of the equine study, scale-up to 100 liters and purification of gram quantities of Cry5B PCC were achieved. These data indicate Cry5B PCC is scalable, highly active, and a potent anthelmintic.

IBaCC, from which PCC is derived, introduced the concept of using a dead Bt bacterium to deliver bioactive Bt Cry proteins. PCC takes that process one step further. In instances where increased purification of Bt Cry proteins is desired (see below), PCC offers significant improvements over other technologies. In addition, in the case of anthelmintic treatment of humans and animals, PCC also offers an attractive pharmaceutical ingredient highly compatible with drug delivery, eliminating the bacterial shell surrounding IBaCC and greatly increasing the purity of the Cry5B protein. We were able to also produce PCC from a strain expressing insecticidal Cry1Ac. The PCC process can thus be extended to a wide range of Bt three-domain proteins. Studies on bioactivity of Cry1Ac PCC against insects are in progress (unpublished data).

Purified Cry proteins have a number of potentially important applications (36). They can be used for crop protection against target insects devoid of live bacteria and bacterial products. This is becoming increasingly important, as there are indications that live Bt spore crystal lysates used as topical sprays might be associated with adverse health events and are subject to increasing regulation. PCC (and IBaCC) are also ideal for releasing improved Cry proteins modified by genetic engineering into the environment. The use of crystals involving release of live recombinant bacteria (spores) is not allowed, since genetically modified genes can spread into other microorganisms. PCC and IBaCC lack this potential danger, since no live bacteria are present, allowing for the release of genetically modified Bt crystal proteins without the potential spread of the modified genes.

Given its purity, PCC is also ideal for making formulations or concentrating Cry protein products to increase specific activity. PCC could be used for producing antibodies for immunoassays for detection of transgenic plants. PCC could also be used in insect and nematode bioassays to determine the effects of a purified Cry protein independent of other potential protein toxins produced by Bt. Importantly, PCC could also be useful for biosafety studies required for use of Cry proteins in transgenic plants and as antiparasitics. Solubilized PCC can be used to study the effects of pure Cry proteins against targets for which uptake of protein potentially requires dissolution of the crystals, e.g., nematodes that restrict intake of large proteins and particles, such as some plant-parasitic nematodes and whipworms (37, 38).

The use of hexane here to process the lysed IBaCC and achieve phase partitioning to aid in crystal purification has also been reported for processing spore crystal lysates and removal of spores (39). The process described here has several advantages over the previous work. First, the PCC process achieves many orders of magnitude greater reduction in CFU per milliliter. Second, the PCC process results in much greater Cry protein purity in protein content, with Cry5B reaching 96% versus 34% of the total protein content for Cry5B PCC and Cry5B IBaCC, respectively.

In summary, we have demonstrated a scalable and simplified procedure to produce a highly purified form of Bt crystals called PCC for use in a diverse range of applications (e.g., drug development, concentration of bioactivity, diagnostics, safety studies, bioactivity studies, formulation for environmental release) where use of purified Bt crystals or soluble Cry proteins are desirable or preferred.

## MATERIALS AND METHODS

### Preparation of PCC.

Bacteria with cytosolic crystal(s) (BaCC) expressing either Cry5B or Cry1Ac were grown and treated with essential oil as previously described to make IBaCC (14). The bacterial cells were lysed using 200 μg/mL lysozyme in buffer (20 mM Tris-HCl [pH 8.0], 2 mM EDTA, 1% Triton X-100) for 2 to 4 h at 37°C. After the lysozyme treatment, the cells were subjected to sonication (small scale) or homogenization (large scale; 15,000 psi, 6 cycles, Avestin Emulsiflex-C3) on ice until 95% bacterial lysis was observed by using a UPlanFl 100× 1.30 numerical aperture oil Ph3 objective on an Olympus BX60 microscope. Soluble bacterial proteins and cellular constituents were removed from the Cy5B crystals by centrifugation at 4,500 rpm for 2 h at 4°C in an Eppendorf 5804R benchtop centrifuge, as the dense crystals are pelletable. Since DNA is a known component of Bt crystals (40–43), an optional DNase treatment can be performed based on the manufacturer’s instructions on the resuspended pellet to remove the nucleic acid.

The crystal-containing pellet was then resuspended in 0.9% saline and emulsified with 25% hexane or food-grade oil (e.g., corn oil) and incubated for 2 h at room temperature in a rotating rotor after pulse sonication to ensure disruption of crystals from cell debris. Following centrifugation, the crystal-containing pellet fraction was washed thrice with sterile water to remove hexane or oil and finally resuspended in sterile water (at a 1/10 volume). These stable PCC aliquots were stored at −80°C and subjected to various quality control analyses and *in vitro* and *in vivo* worm toxicity assays. Microscopy of IBaCC and PCC was carried out using a 100× bright-field microscope. To determine the percent Cry5B protein in IBaCC and PCC, Cry5B IBaCC and PCC were subjected to SDS-PAGE, stained with Coomassie blue, and destained. The gels were imaged using the BIORAD ChemiDoc XRS+ molecular imager system. The intensity of the full-length Cry5B was then compared to the intensity of the protein bands below the full-length Cry5B band. This was done by comparing the integrated density values of the PCC lane and the IBaCC lane. This value was calculated using the most current ImageJ software. This calculation was repeated at various concentrations of IBaCC and PCC, with very similar results obtained with each.

### Animals and parasites.

Ancylostoma ceylanicum and Necator americanus hookworm life cycles were maintained in hamsters as previously described (15) with the exception that N. americanus was maintained with use of 4 mg/liter dexamethasone in the drinking water without any need for supplemental dexamethasone injection. Hamsters were provided with food and water (*ad libitum*). All animal experiments involving hookworms were carried out under protocols approved by the University of Massachusetts Chan Medical School (PROTO202000071/A-2483). All housing and care of laboratory animals used in this study conformed to the NIH Guide for the Care and Use of Laboratory Animals in Research (18-F22) and all requirements and all regulations issued by the USDA, including regulations implementing the Animal Welfare Act (P.L. 89-544) as amended (18-F23). All protocols were approved by the University of Kentucky Institutional Animal Care and Use Committee (protocol 2015-2078) for the equine studies and the USDA Beltsville Institutional Animal Care and Use Committee (17-019) for the murine studies, along with Institutional Biosafety Committee 271.

Egg-to-L3i (infectious third larval stage) assays (Fig. 2) were carried out as described elsewhere (28). For both Fig. 2A and B, the data were normalized to the number of eggs out of ~60 in each well that developed to the L3i stage in the absence of any Cry5B (IBa [14] as the control for IBaCC and water as the control for PCC). For Fig. 2A and 2B, 100% represents between 23 and 27 eggs that developed to L3i, depending upon the negative control and parasite.

Adult hookworms harvested from the intestines of infected hamsters were assayed *in vitro* as previously reported (14), with the exception that motility in mean movement units was read out using the Worminator platform (44) and averaged over 3 min with one adult parasite per well. For *A. ceylanicum*, the data represent the averages of two independent experiments with *n* = 8 adults per dose per experiment. For N. americanus, 8 adults were used in the experimental group and 7 were used in the control. For each *in vitro* experiment, motility was normalized to that of the buffer control at each time point. For *A. ceylanicum* at 8.7 μg/mL, control values were 74.4, 91.9, and 78.5 at 4, 24, and 48 h, respectively. For *A. ceylanicum* at 2.3 μg/mL, control values were 58.5, 82.8, and 74.1 at 4, 24, and 48 h, respectively. For N. Americanus, control values were 78.0, 68.2, 87.2, and 85.3 at 1, 2, 24, and 48 h, respectively.

*In vivo* hookworm curative assays were carried out essentially as described previously (14). For *A. ceylanicum*, hamsters were orally infected with 140 infectious larvae. An overnight collection 18 days postinoculation (p.i.) was taken to determine pretreatment fecal egg counts (FECs). These FECs were used to place hamsters into similarly infected groups. Following grouping, hamsters were weighed and then orally treated with either 0.1 M bicarbonate buffer, 5 mg/kg Cry5B IBaCC mixed with 200 μL of 0.1 M bicarbonate buffer, or 5 mg/kg Cry5B PCC mixed with 200 μL of 0.1 M bicarbonate buffer. Bicarbonate buffer was used to partly neutralize stomach acid and it has no effect on hookworm burdens (14). On day 22 p.i., an overnight collection was taken; on day 23 p.i., animals were weighed and then euthanized. Total intestinal hookworm burdens and FECs were determined, as were changes in body weight. For N. americanus, hamsters were infected subcutaneously with 300 infectious larvae; the curative experiment was otherwise carried out as described previously (14).

Curative studies of *A. suum* infections in mice were carried out similar to those already described (17), except interleukin-13 (IL-13)-knockout mice in a C57BL/6 background were used instead of STAT6-knockout mice. (The IL-13-KO mice were supplied as a gift from the laboratory of Thomas Wynn through an NIH contract with Taconic.) Male and female mice of 3 to 4 months of age were orally inoculated with 1,500 *A. suum* eggs. At 12 days p.i., mice were orally gavaged with either phosphate-buffered saline (control), IBaCC (Cry5B 15 mg/kg), or PCC (Cry5B 15 mg/kg), all as a single dose. At 16 days p.i., mice were euthanized and L4 *A. suum* burdens in the small intestine were assessed.

A pilot curative study in foals was carried out as follows. Two foals naturally infected with *Parascaris* spp. were identified based on fecal egg counts determined by the Mini-FLOTAC technique (45). The timing of weekly fecal egg counts is provided in the figure. One foal was initially treated and the other was kept as a control. To maximize the efficacy, the first foal to be treated was pretreated with 4.0 mg/kg omeprazole (Gastrogard, Boehringer Ingelheim, Ingelheim, Germany) administered orally to neutralize stomach acid and allow more Cry5B to pass into the small intestine. Sixteen hours posttreatment with omeprazole, the foal received PCC via nasogastric tube at a Cry5B dose of 10 mg/kg (~2.5 g/horse). Two weeks after the first foal was removed from the trial, the second foal, which had retained fecal egg counts, was similarly treated with omeprazole and Cry5B PCC.

### Statistical methods.

All statistical analyses and graphs were generated using GraphPad Prism version 9.3.1. All comparisons involving three groups (in which two groups were each compared to a buffer control) were analyzed using a one-way analysis of variance with a one-tailed Dunnett’s posttest to test the hypothesis that treatment would result in a lower worm burden, lower fecal egg count, or increased weight relative to controls. All comparisons between two groups were carried out using a two-tailed Student's *t* test (nonparametric tests such as Mann-Whitney could not be used because of sample size limits, e.g., three hamsters per group in the N. americanus curative experiment).
