# Supplementary material for: Efficient and Scalable Process to Produce Novel and Highly Bioactive Purified Cytosolic Crystals from Bacillus thuringiensis
Source: Microbiol Spectr. 2022 Aug 10;10(4):e02356-22. doi: 10.1128/spectrum.02356-22 (PMC9430767; doi:10.1128/spectrum.02356-22)
Supplement: Supplemental file 1 — Table S1. Download spectrum.02356-22-s0001.pdf, PDF file, 0.02 MB [file spectrum.02356-22-s0001.pdf]

Table S1. Data from rodent *in vivo* experiments

Figure	Treatment	Mean	Standard error
4A	Control	40.3	1.9
	IBaCC	7.4	3.6
	PCC	10.4	3.2
4B	Control	2475	364
	IBaCC	580	231
	PCC	910	222
4C	Control	-0.85	0.61
	IBaCC	3.9	1.3
	PCC	2.3	0.9
5A	Control	8.7	0.7
	PCC	2.4	0.7
5B	Control	2458	50
	PCC	789	50
6	PBS	9.9	1.7
	IBaCC	4.3	1.4
	PCC	4.6	1.2
